# The first child with mixed invasive pulmonary *Mucor* and *Aspergillus* infection: a case report and literature review

**DOI:** 10.3389/fmed.2024.1387278

**Published:** 2024-10-08

**Authors:** Shifu Wang, Shangmin Yang, Jing Ma, Chunyan Zhang, Zheng Li, Mengyuan Wang, Wenwen Yu, Guohua Liu

**Affiliations:** ^1^Department of Microbiology Laboratory, Children’s Hospital Affiliated to Shandong University, Jinan, China; ^2^Shandong Provincial Clinical Research Center for Children’s Health and Disease, Jinan, China; ^3^Department of Ophthalmology, Children’s Hospital Affiliated to Shandong University, Jinan, China

**Keywords:** *Mucor*, *Aspergillus*, pulmonary, coinfection, child

## Abstract

**Purpose:**

Coinfections or consecutive infections of *Mucor* and *Aspergillus* are exceedingly uncommon in children, we report the case to offer the valuable experience for colleagues facing similar situations.

**Case report:**

This report documents the first recorded case of successful treatment for pulmonary mixed infection in a diabetic girl. Initially, the patient underwent treatment based on voriconazole, but the infection continued to deteriorate. Subsequently, bronchoalveolar lavage fluid culture and metagenomic next-generation sequencing (mNGS) were conducted, leading to a clear diagnosis of simultaneous infection by *Aspergillus fumigatus* and *Rhizopus microsporus*. Susceptibility testing revealed fungal resistance to voriconazole. Therefore, a combined treatment regimen of AmB liposomes and isavuconazole effectively eradicated the fungal infection.

**Conclusion:**

This case underscores the importance of early and precise identification of fungal pathogens, determination of effective antifungal medications, and timely implementation of well-planned therapeutic strategies. Furthermore, we comprehensively reviewed 10 cases of pulmonary mixed infections involving *Mucor* and *Aspergillus*, summarizing their characteristics and identifying commonalities.

## Introduction

Invasive pulmonary mucormycosis (IPM) is a rare yet potentially life-threatening opportunistic fungal infection with a high mortality rate ranging from 40 to 80% (1). The clinical presentation of pulmonary mucormycosis, can be challenging to differentiate from that of pulmonary aspergillosis. Common symptoms include fever and cough, while less frequent but possible symptoms such as hemoptysis, pleuritic chest pain, and pleural effusion may also manifest (2). Invasive pulmonary aspergillosis (IPA) is another opportunistic infection that often affects immunocompromised individuals, leading to increased morbidity and mortality (3). Symptoms of IPA typically encompass fever, cough, chest or pleuritic pain, shortness of breath, and/or hemoptysis. In some instances, patients with profound immunosuppression or neutropenia may exhibit minimal or no symptoms due to the absence of an inflammatory response, and suspicion of IPA may only arise after signs of angioinvasion (chest pain or hemoptysis) become apparent (4). Coinfections or consecutive infections of *Mucor* and *Aspergillus* are exceedingly uncommon in children and are predominantly seen in immunocompromised patients (5). Here, we present a case of combined pulmonary infection with *Mucor* and *Aspergillus* in a diabetic girl, with the goal of offering valuable experience to colleagues facing similar situations.

## Case report

Twenty days ago, an 11-year-old girl (40 kg) presented at community hospital with a severe fever and an unexplained cough. Upon diagnosis, she was discovered to have diabetes mellitus. Despite receiving treatment for four days, her symptoms persisted, prompting her to seek further medical care at “Linyi People’s Hospital.” The antibiotics administered included peramivir, meropenem, linezolid, and azithromycin (Linyi People’s Hospital, the exact dose of antibiotics is unknown). On the 11th day of illness, bronchoscopy and alveolar wash were performed, and metagenomic next-generation sequencing (mNGS) identified the presence of *Aspergillus* (151 reads) and *Rhizopus* (131 reads). Subsequently, voriconazole and cefoperazone-sulbactam were added to her treatment regimen (the exact dose of antibiotics is unknown). However, after nine days of therapy, there was no improvement, leading to her transfer to our hospital’s ICU.

**FIGURE 1:**
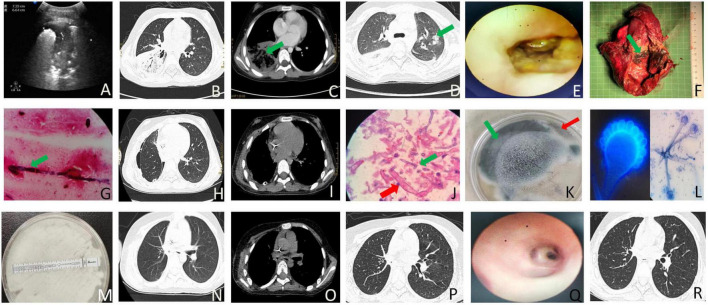
The imaging, laboratory and pathology findings associated with the case. **(A)** B-ultrasound examination of the right lower lung on the dorsal side revealed a solid lung tissue echo measuring 7.3 × 6.8 × 7.1 cm and air bronchial signs on day 2 after admission. **(B)** On day 2 of admission, chest CT showed an air crescent and consolidation in the right inferior lobe. **(C)** Chest CT on day 2 of admission exhibited a reversed halo and consolidation in the right lower lobe. **(D)** A halo-like signature was observed in the left upper lobe on chest CT on day 11 after admission. **(E)** Bronchoscopy on the 12th day of admission revealed mucosa erosion and absence beginning in the middle and lower lobes of the right lung from the middle tracheal opening. The bronchial structure of the distal lobe was destroyed, the lumen was narrow, and a large amount of yellow and white pus moss was observed inside. **(F)** Gross specimen analysis of the postoperative right middle and lower lobes of the lung showed fungal clusters (blue arrow) and several small abscesses (red arrow). **(G)** Lung tissue staining by Gomori Methenamine Silver (GMS) staining showed a small number of fine-branched septate hyphae, angled at 45 degrees. **(H)** Postoperative pulmonary window; chest CT showed right pneumothorax on day 35 after admission following right middle and lower lobe resection. **(I)** Postoperative mediastinal window, chest CT revealed right pneumothorax on day 35 of admission following right middle and lower lobe resection. **(J)** Pulmonary pathology findings identified *Aspergillus* and *Mucor* infections. *Aspergillus* was characterized by fine, 45-degree acute angle, branching septate hyphae (blue arrow, GMS staining, original magnification × 1000), while *Mucor* exhibited aseptate hyphae with right-angled branching (red arrow, PAS staining, original magnification × 1000). **(K)** Culture results indicated the co-growth of *Aspergillus* (red arrow) and Mucor (red arrow). **(L)** Microscopy and fluorescence staining confirmed the presence of Aspergillus (blue arrow) and Mucor (red arrow) in the culture. **(M)** Drug sensitivity testing revealed A. fumigatus resistance to voriconazole. **(N)** Chest CT lung window prior to discharge following right lower and middle lobe resection 2 weeks after discharge. **(O)** Chest CT mediastinal window 2 weeks post-discharge following right lower and middle lobe resection. **(P)** Chest CT lung window 2 weeks after discharge. **(Q)** Bronchoscopy performed 6 weeks after discharge. **(R)** Chest CT lung window observation 6 weeks post-discharge.

Upon admission, the patient’s vital signs were recorded as follows: body temperature, 36.5°C; heart rate, 102 beats/min; respiratory rate, 38 breaths/min; and blood pressure, 131/78 mm Hg. Chest computed tomography (CT) scans revealed air crescent signs, halo signs, and consolidation signs in the right lower lobe. A B-ultrasound examination of the right lower lung on the dorsal side showed a solid lung tissue echo measuring 7.3 × 6.8 × 7.1 cm and air bronchial signs (Figures 1 A–D), leading to a diagnosis of lobar consolidation in the right lung and a small amount of bilateral pleural effusion as indicated by the radiologist. Routine blood tests revealed a white blood cell count of 15.36 × 10^9^/L, a neutrophil count of 14.75 × 10^9^/L, and a C-reactive protein (CRP) level of 276.75 mg/L. Renal function assessments showed a serum creatinine level of 26 μmmol/L (normal range: 27–66 μmmol/L) and a urea nitrogen level of 2.00 mmol/L (normal range: 2.50–6.50 mmol/L). The procalcitonin level was measured at 0.36 ng/ml (normal range: < 0.5 ng/ml). Tests for serum G test (< 37.5 pg/ml, normal range: < 37.5 pg/ml), Candida mannan (< 25.0 pg/ml, normal range: < 25.0 pg/ml) and serum galactomannan (0.215, normal range: < 0.5) returned negative results, while Candida-IgG (259.16 AU/ml, normal range: < 80 AU/ml) and *Aspergillus*-IgG (86.54 AU/ml, normal range: < 80 AU/ml) antibodies were detected as positive. Considering the prior diagnoses of fungal infection and diabetes mellitus, the treatment regimen included insulin, voriconazole (0.325 g, iv, q 12 h), imipenem (600 mg, iv, q 6 h), and linezolid (400 mg, iv, q 8 h).

After three days of treatment, the patient’s condition didn’t get any better. She started throwing up yellow sputum and stomach contents, but the sputum culture came back negative. So, we switched from voriconazole to isavuconazole (200 mg, iv, q 8 h). On the fourth day, her inflammation indicators returned to normal, and her fever had subsided. As a result, she was transferred from the ICU to the respiratory intervention department. She discontinued voriconazole upon admission to the respiratory intervention unit. On the sixth day, a CT scan showed that the right lung lobe consolidation had gotten worse, and there was pleural effusion on the left side. Also, an ultrasound revealed some minor ascites and pelvic effusion. The patient started having sharp pain in the upper left abdomen. To tackle this, we added liposomal amphotericin B (AmB, 150 mg/day). After five days of treatment (on the day 11), the patient was doing better.

On the 12th day, her body temperature improved to 37°C, but she was still dealing with abdominal pain and vomiting light yellow-green fluid. To help ease her symptoms, midazolam, isavuconazole (200 mg, iv, q 8 h) and promethazine were added. The CT scan revealed an interstitial pulmonary parenchymal lesion, bilateral pleural effusion, and fluid build-up in the pelvis. Bronchoscopy showed erosion in the tracheal opening and the middle and lower lobes of the right lung, along with damage to the bronchial structure in the distal lobe, narrowed airway, and a significant amount of yellow and white pus accumulation (Refer to Figure 1E). During the procedure, budesonide (0.5 mg) and epinephrine (1 ml) were administered. After the bronchoscopy, linezolid (400 mg, iv, q 8 h) was replaced with vancomycin (0.5 g, iv, q 8 h).

**FIGURE 2:**
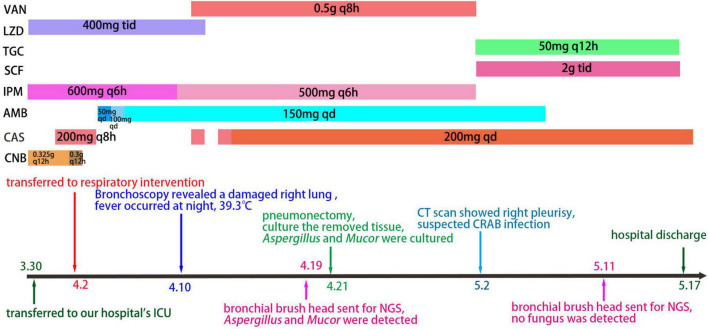
The timeline of data related to patient clinical care. VAN, Vancomycin; LZD, Linezolid; TGC, Tigecycline; SCF, Cefperazone-Sulbactam; IPM, Imipenem; AMB, Amphotericin B; CAS, isavuconazole; CNB, voriconazole.

On the 14th day, she developed low potassium levels, possibly due to insulin and AmB use, so we started giving her potassium supplements. A CT scan showed typical halo signs, lung tissue necrosis, right pleurisy, and fluid in both lungs. On day 23, bronchoscopy and alveolar lavage were performed, and metagenomic next-generation sequencing (mNGS, IDseq™ Ultra fully targeted pathogen capture metagenome by VISION MEDICALS) was conducted. A mixture of pathogens was identified, including *Aspergillus fumigatus* (39 reads), *Rhizopus microsporus* (1 read), Rhizopus oryzae (2 reads), Torque teno virus (46 reads), Human cytomegalovirus (62 reads), and Epstein-Barr virus (9 reads). Surgery was performed to remove the middle and lower lobes of her right lung, with the dissected tissue showing yellow and black necrotic material that was hard (Figure 1F). Histopathological analysis revealed fungal clusters (blue arrow) and small abscesses (red arrow) in the removed lung lobes (Figure 1G). The thoracic and mediastinal windows of CT showed right pneumothorax after right middle and lower lobectomy (Figures 1H, I). The pathologist observed a significant presence of *Aspergillus*, along with a lesser quantity of *Mucor*. This conclusion aligns with the results of the tissue culture (*A. fumigatus* and *Rhizopus microspores* identified by MALDI-TOF MS mass spectrometry, Figures 1J–M). On day 24, she needed mechanical ventilation and chest drainage. Two days later, she was successfully taken off the ventilator and moved to the respiratory care unit. Tracheal tube cultures conducted at the same time identified carbapenem-resistant *Acinetobacter baumannii*, prompting the use of cefoperazone/sulbactam and tigecycline while stopping vancomycin. A follow-up CT scan on day 35 showed improvement in her condition. Tissue culture results on day 37 detected *A. fumigatus* and *Rhizopus microspores*, resistant to voriconazole. Therefore, treatment with isavuconazole (200 mg, iv, q 8 h) was continued. After 12 days of treatment, she was discharged (Figure 2) with prescriptions for faropenem, isavuconazole (200 mg, po, qd), and nifedipine. CT scans conducted at 2 and 6 weeks post-discharge demonstrated ongoing improvement (Figures 1 N–R), with regular parental monitoring advised.

## Discussion

Coinfections or consecutive infections of *Mucor* and *Aspergillosis* are relatively rare, particularly in children. This case report details the first instance of coinfection involving *Aspergillus fumigatus* and *Rhizopus microsporus* in a diabetic girl. *Aspergillus* species are common saprophytic fungi found extensively in the environment, often causing lung infections when their spores or fungal fragments are inhaled (6). *Aspergillus* tends to thrive in cavity lesions, especially within healed tuberculosis cavities in the lungs. In these cavities, the fungus can form mobile fungal balls composed of hyphae (mainly *Aspergillus spp.*, sometimes *Mucor spp*. or a mix of both), inflammatory cells, and fibrin material (7).

Mucormycosis is an infrequent opportunistic fungal infection caused by *Mucor*. Fungal toxins can damage cavity walls and spread locally, resulting in severe hemoptysis, which may be a prominent and often the only symptom of the disease (8). Our patient initially presented with fever and cough, lacking typical lung-related symptoms. It was only after BAL mNGS results indicated *Aspergillus* and *Rhizopus microsporus* infection that she was diagnosed with these dual infections.

Histological examination of lung tissue has long been considered the gold standard for diagnosis, but its implementation is challenging due to compromised respiratory function and an increased risk of bleeding. Therefore, bronchoalveolar lavage (BAL) continues to play a crucial role in the diagnostic process (9). Studies have indicated that compared to testing on BAL samples, serum galactomannan (GM) and β-glucan tests show lower sensitivity and diagnostic odds ratios for diagnosing chronic pulmonary aspergillosis (10, 11). Negative results for GM antigenemia in *Mucor spp*. cases suggest the need for caution to avoid ruling out *Mucor spp.* infection solely based on negative GM testing results (12). In this patient, *Rhizopus microsporus* and *Aspergillus* were identified in the histological examination of lung tissue. However, serum G and serum GM test results came back negative.

Research indicates that risk factors for IPM include diabetes mellitus, metabolic acidosis, iron overload and deferoxamine therapy, immunosuppression, skin or soft tissue injury, broad-spectrum azole use, and other contributing factors (2). Furthermore, studies suggest that viral pneumonia can heighten patients’ vulnerability to secondary bacterial and fungal infections (13). Among the risk factors listed above, our patients only have diabetes, with no other contributing factors present.

We initially prescribed voriconazole to address the infection, but unfortunately, it proved ineffective, likely due to *Mucor*’s resistance to this medication. Subsequently, we administered isavuconazole in combination with AmB, which resulted in an improvement in the patient’s symptoms. Research has shown that AmB has antibacterial properties against both *Mucor* and *Aspergillus* (14). In comparison to voriconazole, isavuconazole was found to be a more effective option against *Aspergillus*. Drug sensitivity tests revealed that the isolated *Aspergillus* fumigatus strain was resistant to voriconazole, often attributed to mutations in the triazoles’ target site (CYP51A) (4). While posaconazole has demonstrated strong efficacy against *Aspergillus spp.* in laboratory settings, its use has primarily been explored for preventing invasive fungal infections in high-risk patients and as a last-resort treatment for stubborn fungal infections (15). Consequently, posaconazole was not included in our treatment plan. Throughout the treatment process, the patient developed hypokalemia due to the use of liposomal AmB and insulin; however, her kidney function remained unaffected, allowing us to continue the treatment regimen. Despite studies indicating that liposomal AmB has reduced nephrotoxicity and lower toxicity in living organisms compared to free AmB (16), it is essential for us to monitor kidney function carefully when administering liposomal AmB.

We searched the PubMed database for cases of coinfections involving *Mucor* and *Aspergillus* in English literature, focusing on pulmonary or pulmonary-plus diseases. Our search identified 10 cases (excluding our patient) from 9 published articles (Table 1). Among these cases, there were 4 female patients and 6 male patients, resulting in a female-to-male ratio of 4:6. The average age was 51.4 years, with an age range of 27 to 70. All patients had infections in the right lung, and 3 also had involvement of the left lung, which may be attributed to the unique anatomy of the right lung. Of the 10 patients, 4 had diabetes mellitus (DM), 2 had undergone organ transplantation, 1 had HIV, 1 had acute myeloid leukemia, 1 had suffered a serious fall injury, and 2 had no known underlying conditions. The average time from symptom onset to diagnosis was 26.8 days (diagnosis timing uncertain for 1 patient). The average hospital stay was 43 days, during which 3 patients passed away, and discharge dates were unclear for 3 patients. Detailed radiographic findings were provided for all patients, with only 3 showing the typical crescent sign and reverse halo sign. The distribution of disease presentation was as follows: 3 patients had bilateral disease, 2 had disease in the upper lobe, 3 had disease in the lower lobe, and 5 had unilateral multilocular disease. Diagnosis was primarily based on histology alone in most cases (6 patients), microbiology alone in 1 patient, and both histology and microbiology in 2 patients. Among the patients, only 1 with acute myeloid leukemia developed the infection while on prophylactic antifungal therapy. The majority of patients (7) received medical therapy alone, while 3 underwent combined medical and surgical treatments; none underwent surgery alone. Six patients received voriconazole therapy, lasting between 1 week and 4 months. Four patients received oral posaconazole therapy, ranging from 4 weeks to 2 months. Seven patients were treated with liposomal AmB, with treatment durations ranging from 1 week to 1 year. Therapy discontinuation was mainly due to developing or fearing acute kidney injury. Surgical interventions were performed when antifungal therapies were deemed insufficient in controlling the disease, typically involving local debridement with wedge resection or lobectomy.

**TABLE 1:** Reportedcases of coinfections between *Mucor* and *Aspergillus* in pulmonary disease.

No	Age	Sex	Comorbidity	Ventilation	Immune	Complication	Antibiotics	Antifungal	CT of Lung
					suppressant				
1 (17)	67	M	ARDS	Y	High-dose steroid (5 weeks)	Kidney and cerebral ischemia	Clindamycin, ganciclovir, levofloxacin	N	Massive infarct pneumonia on both sides
2 (12)	44	M	Renal transplantation	N	Tacrolimus, methylprednisolone tablets	Acute renal failure	Moxifloxacin,	Y	Ground-glass opacification, solid mass opacity with empty opacities, scattering patchy shadows
							valganciclovir		
3 (18)	34	F	HIV	N	CHOP	Meningitis	Ampicillin	N	Ground-glass attenuation, focal consolidations, typical cavitation,
					chemotherapy		cotrimoxazole		
4 (19)	27	F	Diabetes mellitus	Y	/	/	Levofloxacin Cefperazone-Sulbactam	N	Consolidation, cavitation
5 (20)	52	M	Diabetes cardiac transplant	N	Prednisone, tacrolimus,	/	Valganciclovir, trimethoprim- sulfamethoxazole	N	Nodular
6 (21)	58	F	Acute myeloid leukemia	N	Cytarabine, etoposide daunorubicin	Grade 4 diarrhea electrolyte imbalance	Posaconazole	Y	Pleural effusion, consolidation, cavitation, pulmonary abscess
7 (22)	54	F	2 Diabetes mellitus	N	/	/	Levofloxacin	N	Multiple nodules, air crescent signs,
									bilateral pleural effusion,
8 (23)	43	M	High fall injury	Y	/	Hydrothorax	Amoxicillin and clavulanate potassium	N	Scattering patchy shadows
9 (5)	70	M	Hemoptysis fever	N	/	/	/	N	Air crescent signs, right middle lobe fibrosis and traction bronchiectasis
10 (5)	65	M	Cough hemoptysis expectoration	N	/	/	/	N	Reverse halo sign, cavitating consolidation
Right and left	Upper/middle/ lower lobes	Negative	/	/	*Mucor* and *Aspergillus*	42	N	42	Dead
Right and left	Upper lobe	BAL and sequencing showed *Mucor* and *Aspergillus*	+	−	/	19	Voriconazole 1st–15th, intravenous liposomal AmB posaconazole 15th–60th	60	Survival
Right lung	Upper/lower lobes	Negative	/	/	*Mucor* and *Aspergillus*	21	Liposome AmB 22th–42th;posaconazole 43th–103th	/	Survival
Right lung	Upper lobe	Broad right-angled branching aseptate hyphae in BAL	/	/	/	12	Voriconazole 12th–18th	18	Dead
Right and left	Lower lobe	*Aspergillus* fumigatus and septate hyphae	/	+	*Mucor* and *Aspergillus*	60	Caspofungin 60th–88th;voriconazole 60th–165th	88	Dead
Right lung	Middle/lower lobes	Negative	/	/	*Mucor* and *Aspergillus*	45	Posaconazole 3rd–15th day;voriconazole 18th–60th day;liposome AmB 18th–391th day	111	Survival
Right lung	Upper/middle/ lower lobes	Negative	/	+	*Mucor* and *Aspergillus*	29	Voriconazole 23–30th, AmB 30–44th, liposome AmB 45–55th, posaconazole 50th–end	78	Survival
Right lung	Lower lobe	*Mucor* and Aspergillus from BAL	/	−	/	8	Liposome AmB 8th–15th, voriconazole 13th–27th	33	Survival
Right lung	Upper lobe	/	/	/	*Mucor* and *Aspergillus*	21	Liposome AmB 21 days	/	Survival
Right lung	Upper and middle lobes	/	/	/	*Mucor* and *Aspergillus*	/	Intravenous liposomal AmB	/	Survival

Our patient, who had untreated diabetes, initially presented with a prolonged cough and fever. After further investigation, she was diagnosed with a severe mixed infection caused by *Mucor* and *Aspergillus*. The treatment involved an extensive and costly process, ultimately leading to the removal of a significant portion of the middle and lower lobes of the right lung. Fortunately, the prognosis is satisfactory. This case underscores the critical importance of early identification of risk factors for *Mucor* and *Aspergillus*, particularly when related symptoms are present, as it plays a vital role in facilitating timely diagnosis, effective treatment, and improving overall prognosis.

## Conclusion

In conclusion, we have presented the initial instance of a combined *Aspergillus* and *Mucor* infection in a child’s lungs. Precise microbiological and pathological diagnosis, supported by imaging, prompt surgical treatment, interdisciplinary teamwork, and the efficient use of mNGS technology are crucial for effectively addressing lung infections caused by *Aspergillus* and *Mucor* in children. Looking ahead, mNGS technology shows promise for enhancing clinical practices, especially in tailoring treatment approaches accurately for coexisting *Aspergillus* and *Mucor* infections.

## Data Availability

The original contributions presented in the study are included in the article/supplementary material, further inquiries can be directed to the corresponding author.
